# Titanium nanotubes modulate immunophenotyping and cytokine secretion of T cells via IL-17A: a bioinformatic analysis and experimental validation

**DOI:** 10.3389/fimmu.2024.1381158

**Published:** 2025-01-07

**Authors:** Jingju Yin, Yunyang Liao, Shaofeng Liu, Bangwei Che, Hanghang Zhu, Bingbing Yang, Bin Shi

**Affiliations:** ^1^ Department of Oral and Maxillofacial Surgery, The First Affiliated Hospital of Fujian Medical University, Fuzhou, China; ^2^ Oral Medicine Center, National Regional Medical Center, Binhai Campus of The First Affiliated Hospital, Fujian Medical University, Fuzhou, China; ^3^ School of Stomatology, Fujian Medical University, Fuzhou, China; ^4^ Fujian Key Laboratory of Oral Disease, School and Hospital of Stomatology, Fujian Medical University, Fuzhou, China; ^5^ Department of Urology & Andrology, The First Affiliated of Guizhou University of Traditional Chinese Medicine, Guiyang, China

**Keywords:** nanotopography, T cell, immunophenotype, RNA-seq, IL-17A

## Abstract

**Object:**

We aim to explore the immunomodulatory properties of T cells on different titanium nanotubes and the key immunological factors involved in this process.

**Methods:**

Transcriptome data from GEO database of healthy people and healthy implants were used to analyze cell infiltration and factor distribution of adaptive immune using bioinformatics tools. T cells from activated rat were cultured on titanium nanotubes that were prepared by anodization with different diameters (P-0, NT15-30 nm, NT40-100 nm, NT70-200 nm). The proliferation and expressions of the main transcription factors and cytokines of T-cells were detected. Magnetic bead sorting of CD3^+^ T cells and transcriptome sequencing were performed to explore the signaling pathways and key immune factors that may influence the related immune responses.

**Results:**

Bioinformatics analysis showed that healthy peri-implant tissues were enriched by the most of T-cell subtypes. T-cell-mediated adaptive immunological responses involved *IL-17A*. On the third day, the NT15 and NT40 groups showed significantly higher pro-proliferative effects than the NT70 group (*P*<0.05). Notably, the NT40 group exhibited the lowest T-bet expression (*P*<0.05) along with the highest levels of *Rorγt*, *Gata3*, and *Foxp3*(*P*<0.05), followed by the NT15 group. Additionally, the NT40 group demonstrated reduced *RANKL*, *TNF-α*, and *IL-6* (*P*<0.05) and increased *OPG* and IL-10 (*P*<0.05). Meanwhile, the NT15 group had lower *IFN-γ* expression(*P*>0.05) but higher *IL-4*, and *TGF-β1* expressions(*P*<0.05). Differential expressed genes (DGEs) of T-cell related to the morphologies of titanium nanotubes were mostly enriched in the IL-17 signaling pathway mediated by IL-17A/F. Gene and protein expressions indicated that the NT40 group had the highest secretion in IL-17A of T cells.

**Conclusion:**

Titanium nanotube morphologies in medium (100 nm) and small (30 nm) sizes significantly influence T cell differentiation and immune factor secretion, with T-cell-derived IL-17A likely playing a key regulatory role.

## 1 Introduction

The important concept of “osteoimmunology” emphasizes the host immune response in biomaterial-mediated osteogenesis and angiogenesis (1, 2). In the early stage after implantation, macrophages and neutrophils in tissues identify the implants as a foreign body and trigger an initial inflammatory response, which releases many chemokines to activate and induce the migration of monocytes-macrophages, T cells, and dendritic cells (3, 4). As inflammation progresses, B-cell infiltration and T-cell subtype differentiation, which involves CD4^+^ helper T (CD4^+^Th) and CD8^+^ cytotoxic T (CD8^+^Tc) cells, increase (5, 6).

CD4^+^ T cells are important in mediating host immune responses to pathogens, as well as autoimmunity and chronic inflammation. These cells enhance CD8^+^ T-cell responses, promote antibody production in B cells, and influence monocyte/macrophage function (7, 8). TCR/STAT signal mediator transduction and specific cytokine regulation govern the differentiation of naïve CD4^+^ Th cells. Both pathways regulate the expressions of the key transcription factors in each subtype of CD4^+^ Th cells (7, 8), such as *T-bet* (Th1), *Gata3* (Th2), *Rorc* (Th17), and *Foxp3* (Treg), allowing naïve CD4^+^ T cells to differentiate into diverse effector and regulatory cells with distinct cytokine expression profiles, such as *IFN-γ* (Th1), *IL-4* (Th2), *IL-17* (Th17), and *TGF-β* (Treg) (9–11). As suspended cells, T cells rely on these potent cytokine-networks to exert their immune regulatory functions. Studies had shown that Th2, Treg, and Th17 cells or their derived cytokines promote bone regeneration (12–14).

Modifying the topography of implants is an effective strategy to regulate the innate immune response (15, 16), and compared to alteration of the chemical composition of biomaterials, topography optimization is a more durable, stable, and controllable strategy. From the perspective of bionics, the nanoscale structure on the surface of implants is better for osseointegration (17, 18). Electrochemical anodization of pure titanium produces titanium dioxide nanotubes, a common nanotopography that has several advantages, such as a uniform and ordered morphology and control of the diameter and length of nanotubes (19, 20). Previous studies have confirmed that the morphology of titanium nanotube might polarize macrophages toward an anti-inflammatory phenotype that facilitates osseointegration (21, 22). The adaptive immune system, especially T cells and derived cytokines have differently immunomodulatory features, but the morphological effects of nanobiomaterials properties on adaptive immune cells have not yet been determined.

Using bioinformatics data from clinical samples, we explored adaptive immune cell infiltration, especially that of T cells, in healthy peri-implant tissue. Then, T cells were activated and cultured on different titanium nanotubes to explore their immunophenotyping and transcriptomice characteristics. We investigated the potential mechanisms by which titanium nanotubes mediate T-cell specific immune responses.

## 2 Materials and methods

### 2.1 Inclusion and preprocessing of public datasets

The GEO database (https://www.ncbi.nlm.nih.gov/geo/) included gene expression data for healthy implant (HI) and healthy people (HP). We used datasets GSE106090, GSE178351, and GSE57631. The gene expression value was the average of many probes for the same gene symbol after reannotation and normalization. We removed batch effects using the CombAt algorithm of the sva R package and merged datasets with the inSilicoMerging R package (23).

### 2.2 Identification of differentially expressed genes and evaluation of adaptive immune cell infiltration

The limma R package was used to screen DEGs. |logFC|>1 and adjusted *P*<0.05 indicated significant differences. Principal component analysis (PCA) was used to explore distribution differences between HI and HP transcriptome data. Gene set enrichment analysis (GSEA) (v4.2.3) was performed, and the HI and HP gene sets were analyzed for GOBP enrichment. Permutation tests were repeated 1000 times. When *P*< 0.05 and false discovery rate (FDR) < 0.25, the function was regarded as considerably enriched. The TISIDB database (http://cis.hku.hk/TISIDB/) included 341 immune cell marker genes. With the gsva R package for ssGSEA, enrichment scores for 11 T-cell subtypes were calculated based on the expression levels of immune cell-specific marker genes. The findings were visualized using the pheatmap, ggplot2 R packages, and Sanger box platform (http://sangerbox.com/).

### 2.3 Construction of protein network with T-cell-related hub gene and immune factor

DEGs for the HI and HP groups overlapped with 341 T-cell marker genes. Pearson correlation analysis was used to find T-cell-related hub genes from overlapping genes and significantly different enrichment scores of T-cell subtypes. The TISIDB database included 150 immune factors: 24 immunosuppressive factors, 46 immune-stimulating factors, 41 chemokines, 21 major histocompatibility complexes, and 18 receptors. The STRING database (http://string-db.org) was used to construct a protein-protein interaction (PPI) network of T-cell-related hub genes and immune factors, with a confidence level of 0.4. Cytoscape (v3.9.1) was then used for visualization, and the MCODE plug-in was used to identify subclusters of PPI network.

### 2.4 Functional enrichment analysis

With the clusterProfiler R package, Kyoto Encyclopedia of Genes and Genomes (KEGG) enrichment analysis was conducted on DEGs of T cell on titanium nanotubes with different diameters, and a screening criterion of *P*<0.05 was used. An Upset plot was used to visualize the gene intersections when multiple groups were enriched in the same pathway.

### 2.5 Sample preparation and characterization

The pure titanium sheets were obtained from Taiyuxin Metal Materials Co., Ltd., and were ground and polished from 800 to 2000 mesh with silicon carbide sandpapers of two sizes (1×1 cm^2^, 1.5×1.5 cm^2^) and were then ultrasonically cleaned with acetone, absolute ethanol, and first-grade water for 15 minutes each. The smooth surface obtained in this process was used as the control group (P). The samples were acid-etched with 4 wt% HF-5 mol/L HNO_3_ solution. The pure titanium sheet was the anode, the pure platinum sheet was the cathode, and the electrode distance was 1 cm. Glycerol with 0.5 wt% NH_4_F and 10 vo1% H_2_O was the electrolyte. Magnetic stirring was maintained at 30°C, and a DC-regulated power supply powered it. The time was 4 hours. The NT15, NT40, and NT70 titanium nanotubes with 30, 100, and 200 nm diameters were synthesized at 15 V, 40 V, and 70 V voltages, respectively, and annealed at 450°C for 2 hours. The autoclave was prepared for use.

The surface morphology, roughness, and hydrophilicity of different samples were characterized using field emission scanning electron microscope (FE-SEM) (Zeiss Sigma 300, Germany), atomic force microscope (AFM) (Dimension Icon, Bruker, Germany), and contact angle goniometer (DSA-25E, Krüss, Germany), respectively.

### 2.6 Cell culture and identification

Sprague-Dawley rats (male, 6-8 weeks, 200-220 g) were obtained from the SLAC Experimental Animal Co., Ltd, Shanghai, China. The spleens of Sprague-Dawley rats were aseptically separated, and the cell suspensions of spleens were prepared. The density gradient centrifugation was used to obtain the second layer of white flocculent cells in the middle, which were lymphocytes (24–26), and it was then cultured in a T25 flask using RPMI 1640 medium and 10% fetal bovine serum (Hyclone, USA) in humidified air with 5% CO_2_ at 37°C. This study was approved by the Experimental Animal Ethical Review Committee of Fujian Medical University (IACUC FJMU 2023-0191).

The concentrations of rat splenocyte and lymphocyte were adjusted to 10^6^ cells/mL. The cells were incubated with or without APC anti-rat CD3 (BD Pharmingen, USA) and PE anti-rat CD45RA (Biolegend, USA) antibodies on ices in the dark for 20 minutes and were washed twice with PBS. The flow cytometry identified CD3 and CD45RA cell surface markers. Data analysis was performed using FlowJo_v10.6.2.

### 2.7 Carboxy fluorescein diacetate succinimidyl ester labeling and activated T-cell subtypes

After removal, lymphocytes were resuspended in DPBS with 5% heat-inactivated fetal bovine serum. The suspensions were mixed with CFSE (5μM, BD Pharmingen, USA) by vortexing at low speed. The lymphocytes were labeled at room temperature for 10 minutes in the dark, washed with DPBS, and placed in a T25 flask to grow.

A 12-well plate was precoated with PBS (1μg/mL, CD3 monoclonal antibody, Thermo Fisher Scientific, USA) for 24 hours at 4°C. Lymphocytes were then seeded on the plate. The CD28 monoclonal antibody (5μg/mL, Thermo Fisher Scientific, USA) was added to target T-cell activation. The CFSE fluorescence intensity was detected by flow cytometry after 3 days of dark culture. Data analysis was performed using FlowJo_v10.6.2.

Each group was cultivated for 3 days with activated T-cells. The sample size was 1×1 cm^2^ for 24-well plate and 1.5×1.5cm^2^ for 12-well plate. Cells seeded in 24-well plates were used for the CCK8 experiment and RT-qPCR, whereas magnetic bead sorting, transcriptome sequencing, and ELISA were performed in 12-well plates. The concentrations of cultured cells were all 4×10^6^ cells/mL.

### 2.8 Cell proliferation assay

The CCK8 solution (Dojindo, Japan) was added to each well and incubated for 2 hours at 37°C after 1 to 3 days of cell growth. The cell suspensions were collected into a 1.5 mL EP tube and centrifuged at 350g for 5 minutes. The supernatant from each tube was aspirated at 110 μL and transferred to a new 96-well plate. The OD value was measured at 450 nm.

### 2.9 RT-qPCR

Total RNA was extracted with RNAiso Plus reagent (TaKaRa, Japan), and reverse transcription was performed with the PrimeScript™ RT reagent Kit (TaKaRa, Japan). TB Green Premix Ex Taq (TaKaRa, Japan) was used for RT-qPCR on a LightCycler480 (Roche, Germany). The data were subjected to the comparative cycle threshold method (ΔΔCt) and normalized to a housekeeping gene, *GAPDH*. The primer sequences for the key transcription factors of T-cell subtypes, such as *T-bet* (Th1), *Gata3* (Th2), *Rorγt* (Th17), *Foxp3* (Treg), and the cytokines *RANKL*, *OPG*, *VEGFA, ANG-2, HIF-1α*, *IL-4*, *IL-10*, *TGF-β1*, *IFN-γ*, *TNF-α*, *IL-6* and *IL-17A* are shown in **Supplementary Table 2**.

### 2.10 Magnetic bead sorting and transcriptome sequencing of T cells

The cell concentration was adjusted according to the EasySep™ Rat T-Cell Isolation Kit (STEMCELL, Canada) instructions for CD3^+^ T-cell negative selection. The sorted cell pellets were added to 1mL of RNAiso Plus to completely dissolve. The samples were prepared for RNA extraction, quality inspection, and UID-library construction. Then, the NovaSeq 6000 (Illumina, USA) platform was used for sequencing.

### 2.11 ELISA

Following the ELISA kit (NeoBioscience, China) instructions, the fitting curve of the quadratic polynomial equation was drawn with the standard concentration as the ordinate and OD as the abscissa. The *IL-17A* concentration was calculated in the supernatants of each sample under the standard curve.

### 2.12 Statistical analysis

The data presented as the mean ± standard deviation and were analyzed using SPSS 23.0 (IBM, USA) and visualized using Prism 9.3.0 (GraphPad Software, USA). Multiple comparisons were analyzed by the One-way ANOVA, followed by LSD-t test for parametric data and the Kruskal-Wallis test for nonparametric data. Values of *P*< 0.05 were considered statistically significant.

## 3 Results

### 3.1 Removal of the batch effect

The sample distributions of the GSE106090, GSE57631, and GSE178351 datasets differed substantially before the batch effect was removed (**Supplementary Figures 1A, C, E**). After eliminating the batch impact, the data distribution remained consistent. The boxplots showed that the median was on a straight line (**Supplementary Figure 1B**), the density plot showed that each dataset curve was substantially fitted (**Supplementary Figure 1D**), and the Umap chart showed that the samples in each dataset were distributed and interlaced with one another (**Supplementary Figure 1F**). This indicated that the batch differences were eliminated.

### 3.2 PCA and DEGs analysis

**Figure 1:**
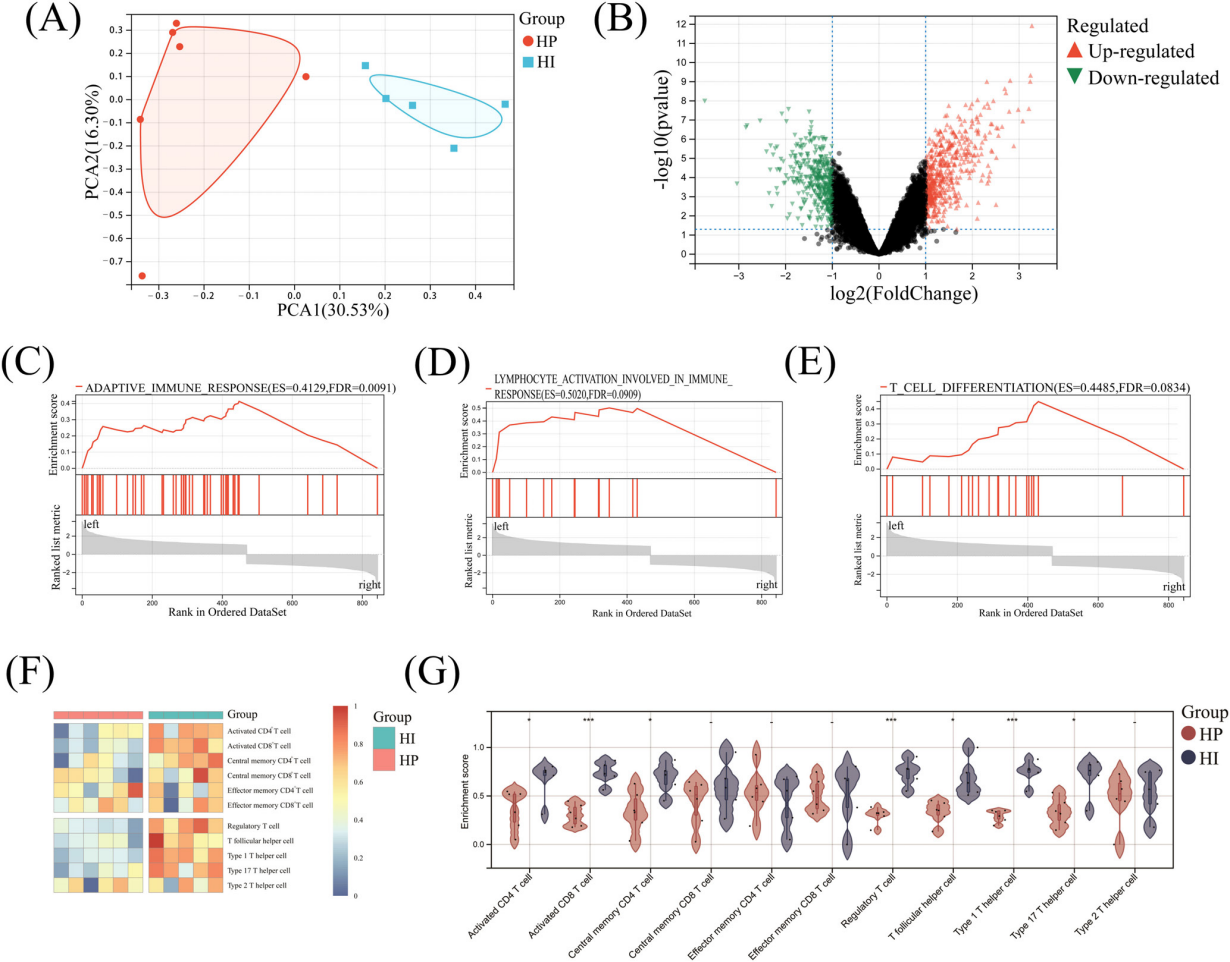
Comprehensive analysis of DEGs and T cells in healthy implants versus healthy people. **(A)** Principal component analysis; **(B)** Volcano map; **(C-E)** GSEA enrichment analysis; **(F)** Heatmap of the landscape of T cell infiltrations; **(G)** Violin diagram. ^-^ No significance, ^*^ *P*< 0.05, ^***^ *P*< 0.001.

PCA analysis demonstrated a pronounced separation trend between the HI and HP groups, highlighting distinct characteristics of gene expressions in the two sample cohorts. The first principal component (PC1) accounted for 30.53% of the variance, while the second principal component (PC2) explained 16.30%, collectively underscoring significant differences in gene expression profiles between healthy implants and healthy individuals (**Figure 1A**). Furthermore, the volcano plot illustrated the distribution of DEGs between the two groups, identifying 470 up- and 374 down-regulated DEGs in the HI group compared to the HP group (**Figure 1B**). These results reveal substantial genetic divergence in clinical tissue samples from the HI and HP groups.

### 3.3 Adaptive immune cell infiltration

GSEA of HI and HP mRNA expression matrices revealed significant enrichment in adaptive immune response (ES=0.4129, FDR = 0.0091), lymphocyte activation (ES=0.5020, FDR = 0.0909), and T-cell differentiation (ES=0.4485, FDR = 0.0834) in the HI group, with all values below 0.25 and *P*<0.05 (**Figures 1C–E**). This result showed that there was indeed a high degree of adaptive immune response enrichment in the HI group, and it was significantly related to T-cell activation and differentiation.

The heatmap indicated that squares in the HP group were mainly cold colors, suggesting a low abundance of T-cell infiltration, whereas squares in the HI group were mostly warm colors, indicating a high abundance (**Figure 1F**). In the HI group, 7 T-cell subtypes (Activated CD4^+^ T, Activated CD8^+^ T, Central memory CD4^+^ T, Regulatory T, T follicular helper, Type 1 T helper, and Type 17 T helper cells) were significantly higher than those of the HP group (*P*<0.05), while other cells such as Type 2 T helper, Central memory CD8^+^ T, Effector memory CD4^+^ and CD8^+^ T cells showed no significant difference between the two groups (*P*>0.05)(**Figure 1G**).

### 3.4 The protein interaction network with hub genes of T cells and immune factors

**Figure 2:**
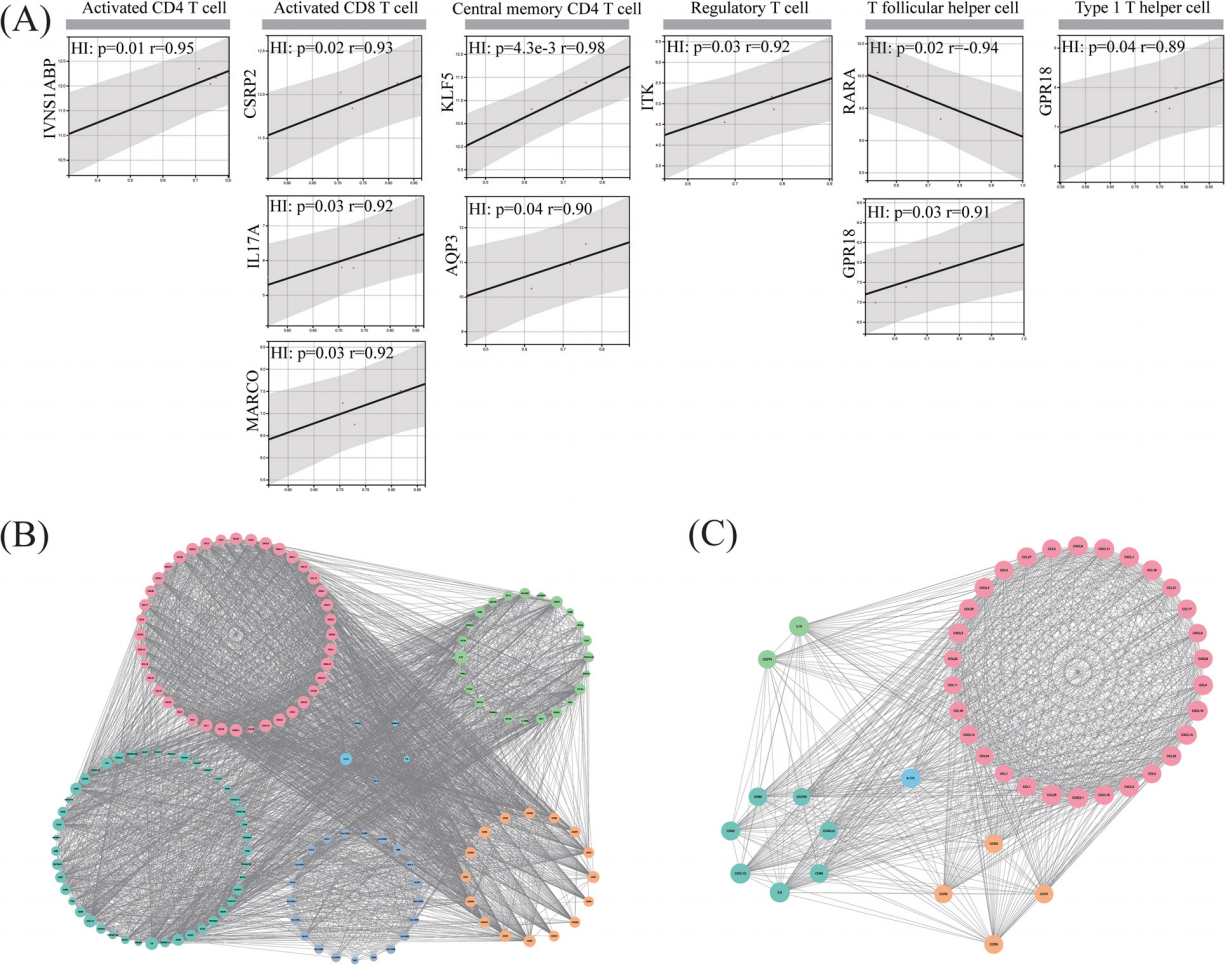
Comprehensive analysis of T-cell-related hub genes. **(A)** Pearson correlation scatter plot of 9 T-cell-related hub genes with 7 differential T cells; **(B)** PPI network diagram of T-cell-hub genes and immune factors; **(C)** Important subclusters of IL-17A, which is the core. Blue, light green, dark green, orange and pink represented T-cell-related hub genes, inhibitory factors, stimulatory factors, receptors, and chemokines, respectively.

DEGs from the HI and HP groups overlapped with 341 T-cell-specific marker genes, obtaining 36 genes. Pearson correlation analysis was conducted on the overlapping gene expression levels and the enrichment scores of 7 substantially distinct T-cell subtypes (Activated CD4^+^ T, Activated CD8^+^ T, Central memory CD4^+^ T, Regulatory T, T follicular helper, Type 1 T helper, and Type 17 T helper cells) to identify 9 T-cell-related hub genes (*IVNS1ABP, CSRP2, IL-17A, MARCO, KLF5, AQP3, ITK, RARA, GPR18*) (**Figure 2A**).

T-cell-related-hub genes and 150 immune factors were included in the STRING database, and only *ITK*, *RARA*, *KLF5*, *IL-17A*, and *MARCO* participated in the PPI network (**Figure 2B**). The MCODE plug-in identified 6 network subclusters (**Supplementary Figure 2**), with just one overlapping hub gene, *IL-17A*, in the highest-scoring subcluster (**Figure 2C**). Based on the number of genes participating in the network subclusters, we discovered the chemokines dominated this subcluster, followed by immunological stimulatory factors, receptors and inhibitory factors.

### 3.5 Preparation and characterization of titanium nanotubes

**Figure 3:**
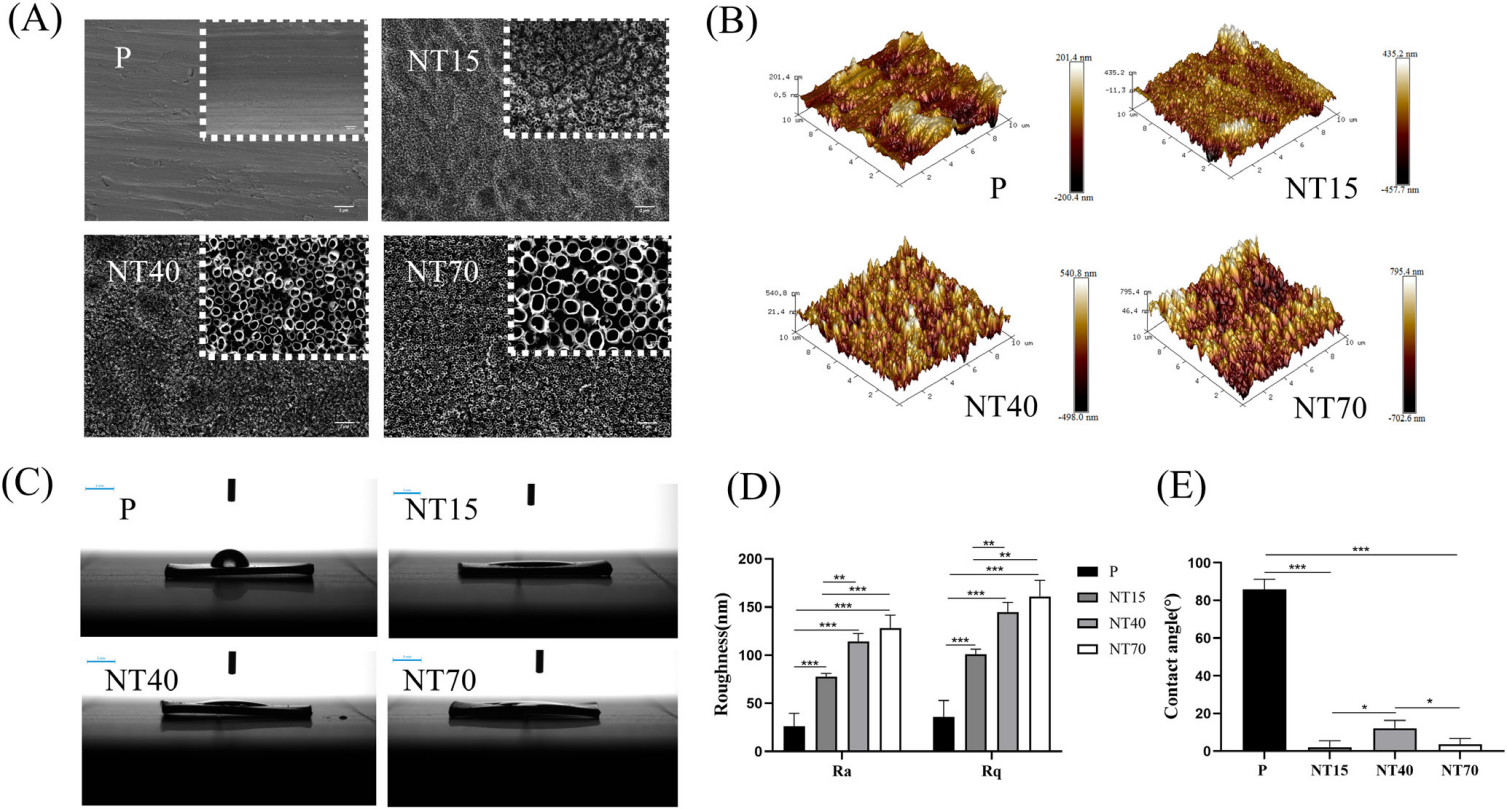
Characterization of the morphology of titanium nanotubes in terms of physical and chemical characteristics. **(A)** Diagram of scanning electron microscopy (scale bars: 200 nm and 2 μm). **(B)** 3D roughness chart; **(C)** Deionized water test chart (scale bar: 3 mm); **(D)** Roughness and **(E)** Contact angle comparison chart. n=3. Ra and Rq data conformed to the normal distribution and homogeneity of variances, One-way ANOVA. Contact angle data did not conform to the normal distribution, Kruskal-Wallis test. ^*^ *P*<0.05, ^**^ *P*<0.01, ^***^ *P*<0.001.

The titanium nanotubes were generated at 15V, 40V, and 70V voltages with diameters of 30.59 ± 4.99nm, 102.95 ± 7.64nm, and 203.47 ± 8.70nm, representing small (NT15-30nm), medium (NT40-100nm), and large (NT70-200nm) diameters, respectively. The electron microscope showed a uniform tubular structure without collapse or broken tubes (**Figure 3A**). The Ra and Rq values showed that as the diameter of the titanium nanotube increased, the roughness increased and was significantly higher than that of the P group (*P*<0.05) (**Figures 3B, D**). Titanium nanotube surfaces had considerably lower contact angles than the P group (*P*<0.05), followed by the NT40 group which has higher contact angles than the NT15 and NT70 groups (*P*<0.05). (**Figures 3C, E**). It was indicated that the NT15 and NT70 groups had the best hydrophilicity. **Supplementary Table 1** listed the detailed data of roughness and contact angle for each group.

### 3.6 Extraction of rat lymphocytes

**Figure 4:**
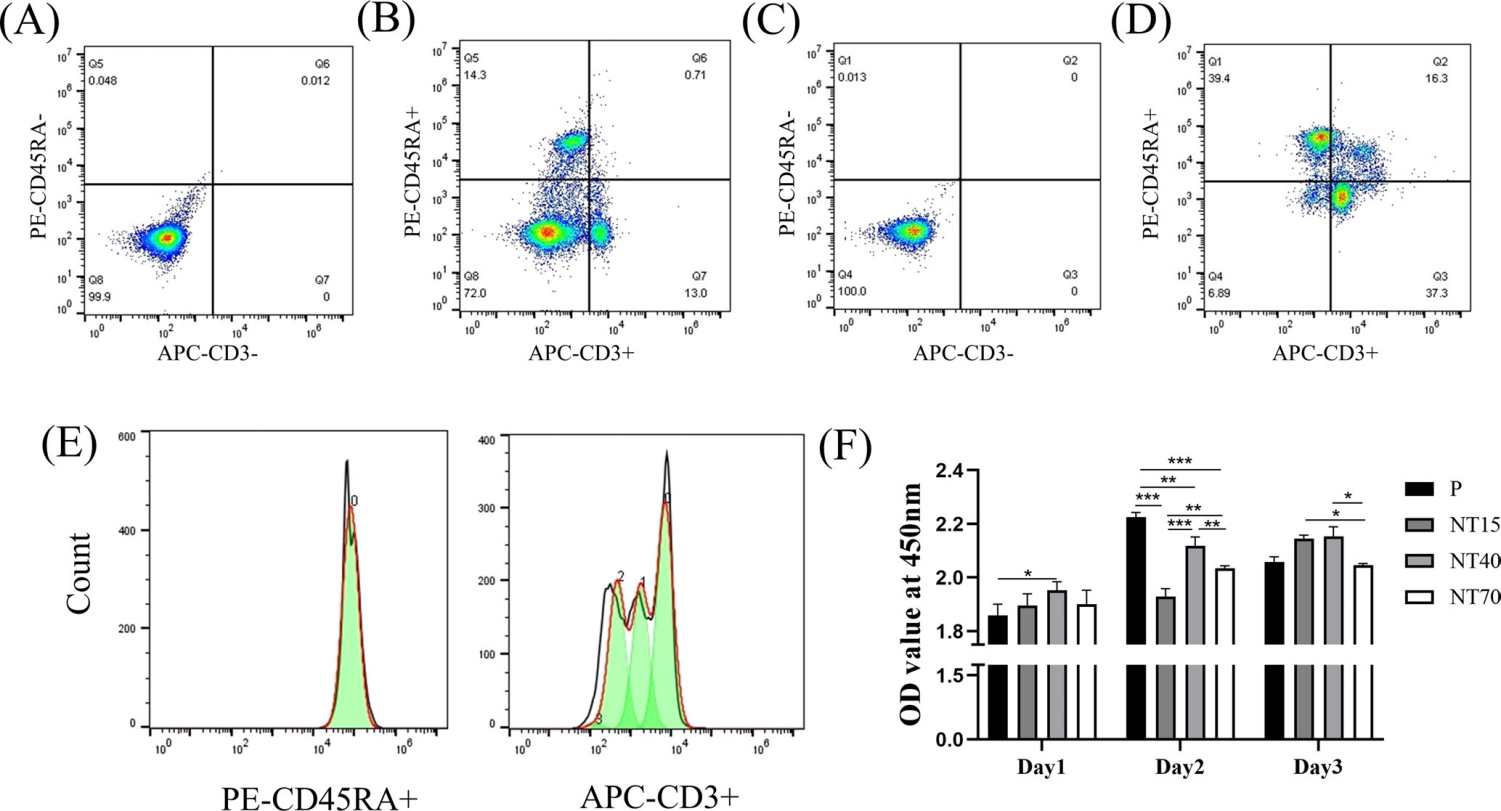
Extraction, activation and flow cytometry analysis of cells. The proportions of T and B cells in splenocytes with **(A)** antibody-negative and **(B)** antibody double-staining. The proportion of T and B cells were extracted by density gradient centrifugation in lymphocytes with **(C)** antibody-negative and **(D)** antibody-double staining. **(E)** The proliferation peak diagrams of B and T cells were labelled by CFSE; **(F)** The proliferation abilities were compared among the P, NT15, NT40, and NT70 groups. n=3. The OD values of Day1 and Day2 conformed to the normal distribution and homogeneity of variances, One-way ANOVA. The OD value of Day3 did not conform to the normal distribution, Kruskal-Wallis test. ^*^ *P*<0.05, ^**^ *P*<0.01, ^***^ *P*<0.001.

T and B cells comprised 14.3% and 13.0% of splenocytes before lymphocyte separation (**Figures 4A, B**). After density gradient centrifugation, T and B cells comprised 37.30% and 39.40% of lymphocytes, respectively. Overall, non-lymphocytes were reduced to 6.89%, while double stained cells, T and B cells increased to approximately 80% (**Figures 4C, D**). The results demonstrated that this method is effective for purifying lymphocytes.

### 3.7 Activation of T cells *in vitro*

T cells, but not B cells, which had no apparent peak, showed an apparent proliferation peak on the third day under CFSE fluorescent labeling (**Figure 4E**), demonstrating that CD3/CD28 specifically stimulated T-cell proliferation. For CCK-8 proliferation assay, the titanium nanotube group demonstrated a temporal gradient of T-cell proliferation. On the first day, the NT40 group had significantly higher T-cell proliferation than the P group (*P*<0.05). And, the NT40 group still had the best pro-proliferation activity, followed by NT15 group, both of which were significantly higher than NT70 group on the third day (*P*<0.05) (**Figure 4F**).

### 3.8 T-cell immunophenotyping

**Figure 5:**
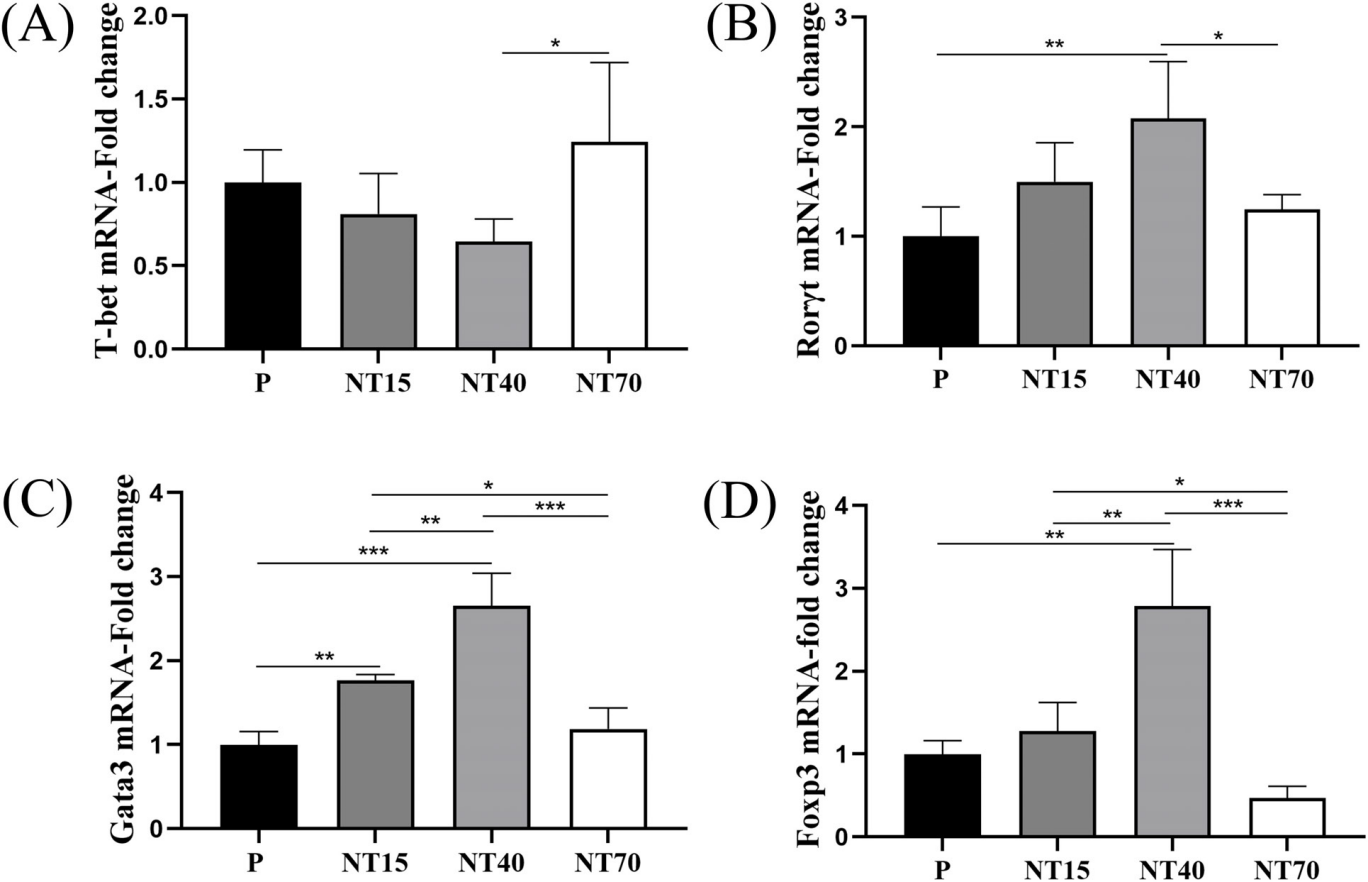
Expression levels of transcription factors of T cell subtypes in the P, NT15, NT40, and NT70 groups. **(A-D)** Comparison of the expression levels of T-cell transcription factors were compared. n=3. All data conformed to the normal distribution and homogeneity of variances, One-way ANOVA. ^*^ *P*<0.05, ^**^ *P*<0.01, ^***^ *P*<0.001.

We evaluated the gene expressions of *T-bet*, *Gata3*, *Rorγt*, and *Foxp3*, which are transcription factors for Th1, Th2, Th17, and Treg subtypes. The results showed that T cells in the NT40 group had the lowest *T-bet* gene expression and the highest gene expression in the NT70 group (NT40 vs NT70, *P*<0.05) (**Figure 5A**). Regarding the expression levels of *Rorγt*, *Gata3*, and *Foxp3*, the NT40 group was the most able to promote the expressions of these transcription factors (vs P group, *P*<0.05), followed by the NT15 group, with the P and NT70 groups having the lowest expression levels (**Figures 5B–D**).

### 3.9 Cytokine secretion of T cells

**Figure 6:**
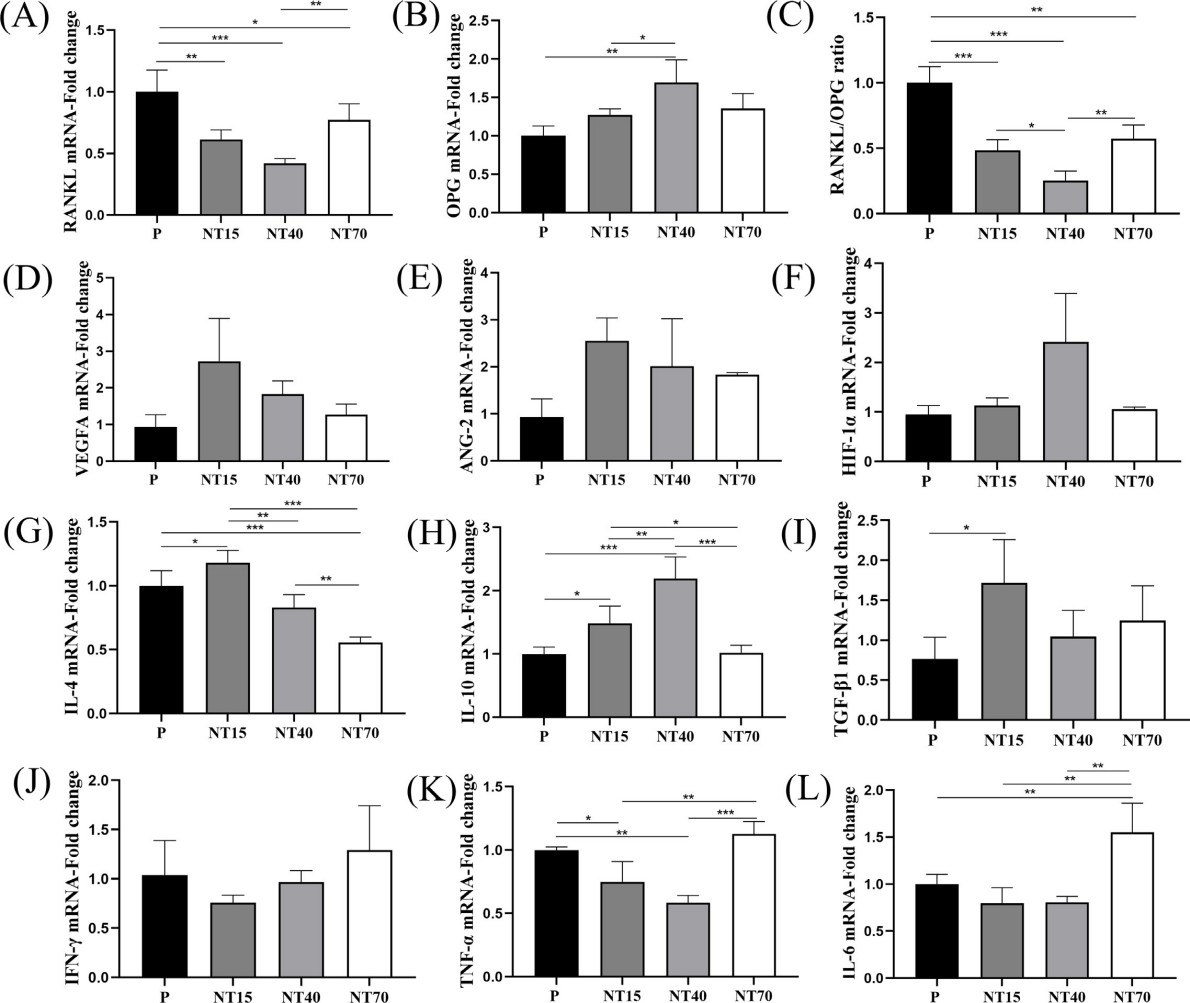
The expression levels of T-cell cytokines were compared among the P, NT15, NT40, and NT70 groups. **(A–C)** Osteoblastic and osteoclastogenic genes. **(D–F)** Angiogenesis genes. **(G–I)** Anti-inflammatory genes. **(J–L)** Pro-inflammatory genes. n=3.The data of VEGFA, ANG-2, HIF-1α did not conform to the homogeneity of variances, Kruskal-Wallis test. The rest of genes'data conformed to the normal distribution and homogeneity of variances, One-way ANOVA. *P<0.05, **P<0.01, ***P<0.001.

The titanium nanotube morphology significantly inhibited *RANKL* expression, with the NT40 group having the best inhibitory ability (vs P and NT70, *P*<0.05), followed by the NT15 group (vs P, *P*<0.05). The NT40 group also showed significantly higher expression of osteogenic factor *OPG* compared to the NT15 and P groups (*P*<0.05). The *RANKL/OPG* ratio more intuitively showed the best bone-promoting effects of the NT40 group (**Figures 6A–C**). The expression of *VEGFA* and *Ang-2* on the NT15 group was higher than P group, followed by the NT40 group, but there was no statistical difference. The expression of *HIF-1α* in the NT40 group was higher than other three groups, with no statistical difference (**Figures 6D–F**).

For anti-inflammatory factors, it observed that the NT15 group had considerably highest *IL-4* expression level than the other three groups (*P*<0.05), whereas the NT70 group had the lowest expression (*P*<0.05). *TGF-β1* was also considerably higher in the NT15 group compared to the P group (*P*<0.05). And the *IL-10* was significantly highest in the NT40 group compared to the other three groups (*P*<0.05), followed by the NT15 group (*P*<0.05) (**Figures 6G–I**). For pro-inflammatory factors, compared to the P group, the NT15 and NT40 groups demonstrated inhibited expressions of the *IFN-γ*, *TNF-α*, and *IL-6*, whereas the NT70 group significantly enhanced them (**Figures 6J–L**).

### 3.10 DEGs of actived T-cells

**Figure 7:**
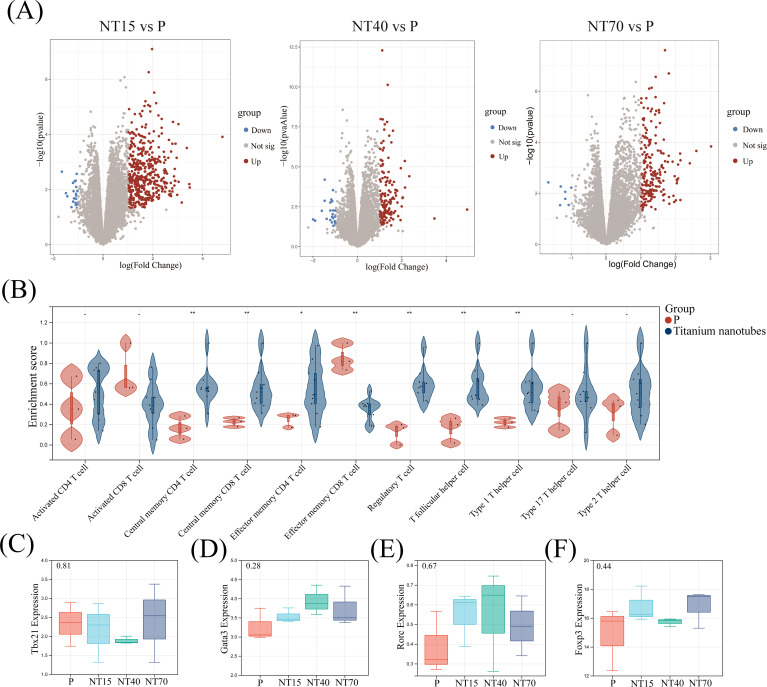
Comprehensive analysis of DEGs, T-cell infiltration and the expressions of major transcription factors were derived from the transcriptome sequencing data. **(A)** Volcano plots of DEGs in the NT15, NT40, and NT70 groups vs. the P group; **(B)** Violin diagram; **(C-F)** Boxplots. n=3. The expressions of *Tbx21*, *Gata3*, and *Rorc* conformed to the normal distribution and homogeneity of variances, One-way ANOVA. The expression of *Foxp3* did not conform to the normal distribution, Kruskal-Wallis test. ^-^ No significance, ^*^ *P*<0.05, ^**^ *P*<0.01.

After magnetic bead sorting of CD3^+^ T cells on titanium nanotubes of various diameters and smooth surface, RNA-seq was performed, and the results showed that the NT15, NT40, and NT70 groups had 452 (429 up- and 23 down-regulated genes), 164 (141 up- and 23 down-regulated genes), and 209 (202 up- and 7 down-regulated genes) DEGs, respectively, compared to the P group (**Figure 7A**).

### 3.11 Analysis of T-cell sequencing data with the morphology of titanium nanotube

A comparison of the infiltration of 11 T-cell subtypes showed that the morphologies of titanium nanotubes significantly promoted T-cell subtype differentiation. Only Activated CD8^+^ T cells and Effector memory CD8^+^ T cells were considerably higher in the P group compared to the group of titanium nanotube morphology (*P*<0.05). The remaining 9 T-cell subtypes were all more infiltrated in the nanotube group, among which Central memory CD4^+^T, Central memory CD8^+^T, Effector memory CD4^+^T, Regulatory T, T follicular helper and Type 1 T helper cells were significantly higher than those in the P group (*P*<0.05)(**Figure 7B**). We collected the expressions of *Tbx21* (*T-bet*), *Rorc* (*Rorγt*), *Gata3*, and *Foxp3* from transcriptome sequencing data of the P, NT15, NT40, and NT70 groups. **Figures 7C–F** showed that the expression levels of the *Tbx21* (*T-bet*), *Rorc* (*Rorγt*), and *Gata3* genes were consistent with the results from RT-qPCR (*P*> 0.05).

### 3.12 KEGG pathways

**Figure 8:**
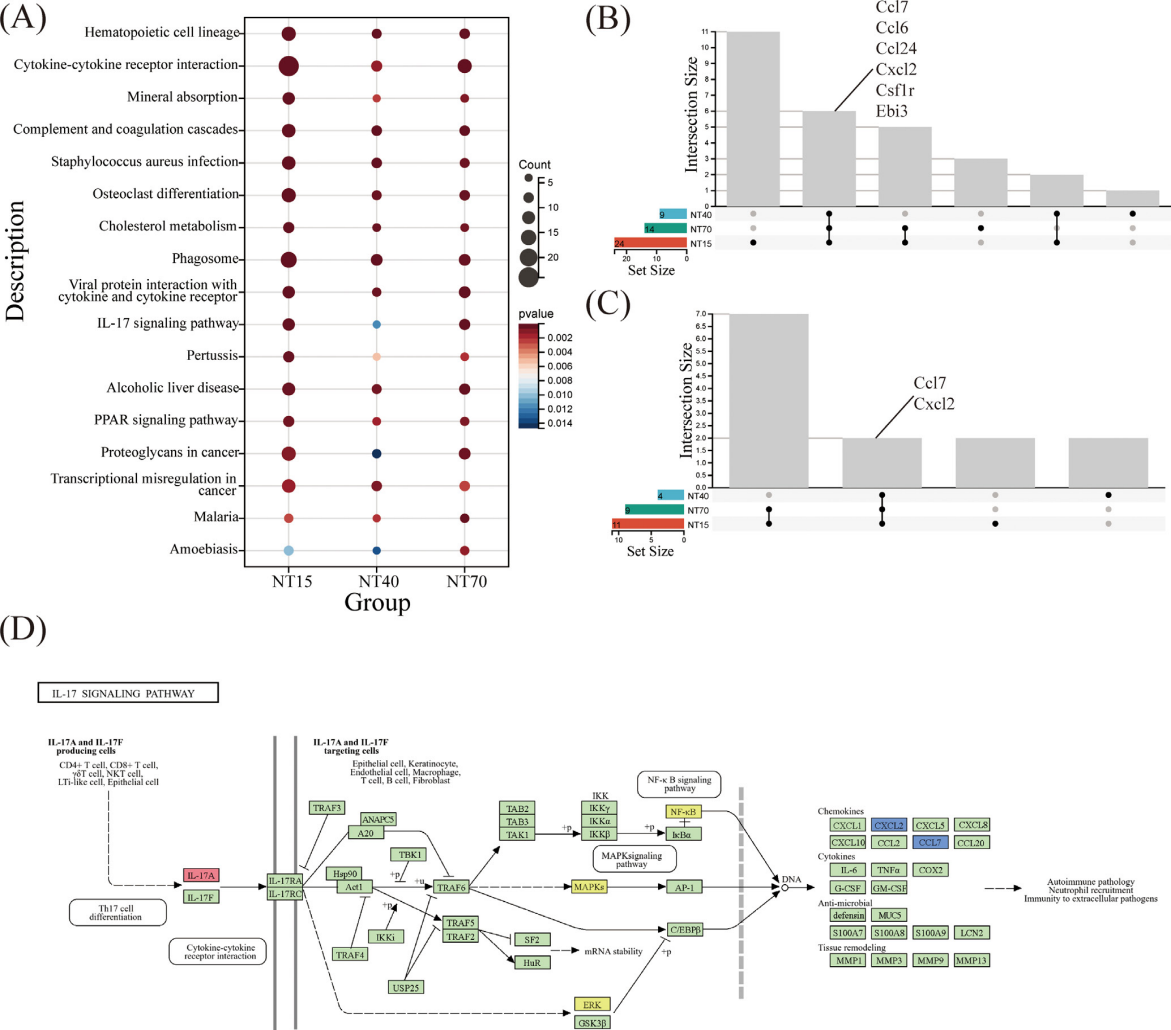
Comprehensive analysis of KEGG pathways in the NT15, NT40, and NT70 groups vs. the P group. **(A)** 17 KEGG-enriched pathways were common to the three groups. Three groups of DEGs were co-enriched in **(B)** Cytokine-cytokine receptor interaction and **(C)** IL-17 signaling pathways; **(D)** A schematic diagram of the IL-17A/F KEGG pathway (https://www.kegg.jp/pathway/map=map04657&keyword=IL-17A).

KEGG enrichment analysis revealed that NT15, NT40, and NT70 groups all detected 17 shared signaling pathways among the top 30 biological activities. These pathways include Hematopoietic cell lineage, Cytokine-cytokiner receptor interaction, Complement and coagulation cascades,etc. Such findings suggested that titanium nanotubes may modulate immune system functions by activating these essential molecular pathways (**Figure 8A**). To delve deeper, we investigated the overlapping gene differences in the NT15, NT40, and NT70 groups, focusing specifically on the Cytokine-cytokine receptor interaction and IL-17 signaling pathways which relate to immunological function. Within the cytokine-cytokine receptor interaction pathway, differential expression was identified for Ccl7, Ccl6, Ccl24, Cxcl2, Csflr, and Ebi3 across the three groups. Similarly, in the IL-17 signaling pathway, Ccl7 and Cxcl2 emerged as key DEGs. These findings indicate that Overlapping Ccl7 and Cxcl2 might serve as critical chemokines through which titanium nanotubes regulate T-cell-mediated immunity (**Figures 8B, C**).

The KEGG pathway analysis showed that *Ccl7* and *Cxcl2*, two key chemokines, were involved in the IL-17 branch signaling pathway mediated by *IL-17A/F*, which regulates tissue remodeling via downstream ERK and NF-_K_B signaling targets (**Figure 8D**). Also based on the bioinformatics analysis of HI and HP samples (**Figures 1**, **2**), *IL-17A* might be a key immunological factor in T cells.

### 3.13 IL-17A measurement

**Figure 9:**
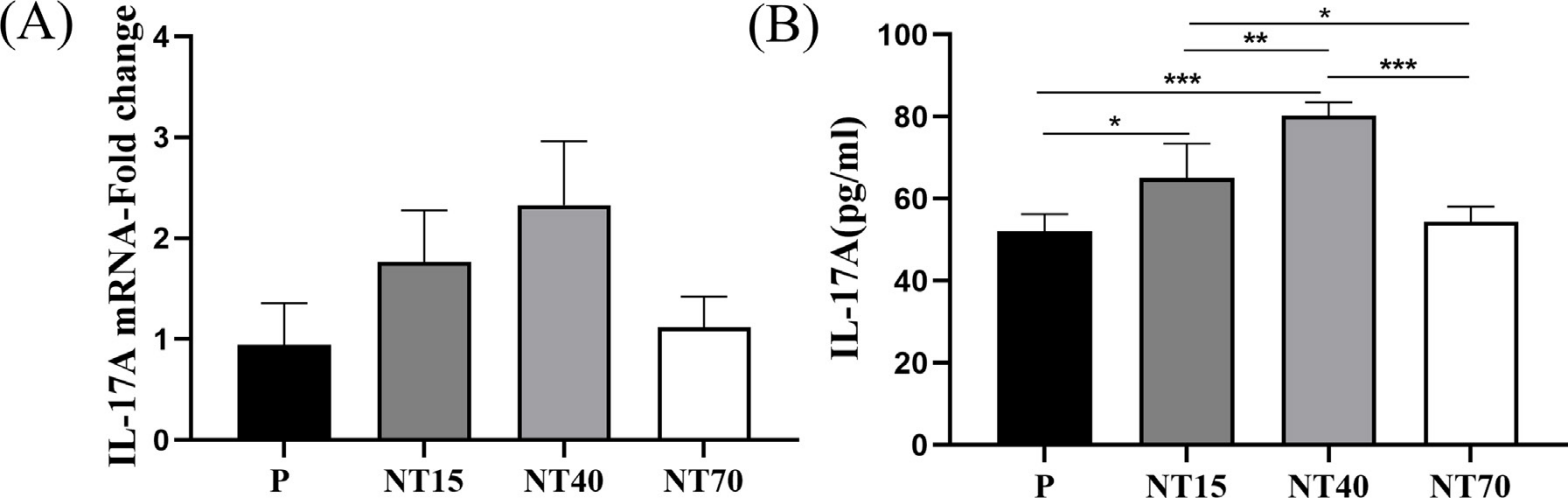
The gene and protein expressions of IL-17A in T cells were compared among the P, NT15, NT40, and NT70 groups. **(A)** RT-qPCR; **(B)** ELISA. n=3. RT-qPCR data did not conform to the normal distribution, Kruskal-Wallis test. ELISA data conformed to the normal distribution and homogeneity of variances, One-way ANOVA. ^*^ *P*<0.05, ^**^ *P*<0.01, ^***^ *P*<0.001.

To confirm the differences in the target protein IL-17A released by T cells following activation of titanium nanotubes of varying diameters, we performed RT-qPCR and ELISA tests and discovered a consistent trend. **Figure 9** showed that the T cells in the NT40 group secreted the highest concentration of IL-17A on the morphology of 100-nm titanium nanotubes, followed by the NT15 group. And the groups P and NT70 generated the least IL-17A. The ELISA findings are significant.

## 4 Discussion

Studies suggest that adequate implant osseointegration requires a proper immune microenvironment (27, 28). Most investigations have focused on favorably osseointegrative effects of the material surface on macrophage polarization (4, 29). As for T cells, which are suspended, have less cytoskeleton and pseudopodia plasticity than macrophages and therefore have received less attention. In this study, we aimed to explore how titanium nanotubes of various diameters affect the immune activity of T cells and the essential immunological factors involved.

Implants may harm bone tissue when placed as foreign entities. Chemotaxis, migration, and activation of immune cells result from damage-associated molecular patterns (DAMPs) release and immune system activation (4). A previous study revealed that diverse immune cell heterogeneity and dynamic changes are modulated by implant properties (30). Among these immunological cells, T cells that mediate adaptive immunity may cluster near the sites of biomaterial implantation and differentiate into diverse subtypes to contribute to macrophage polarization (31). This process was identified via bioinformatics analysis of public datasets in our research. The osseointegration of healthy implant is enriched in T-cell activation and differentiation, including Activated CD4^+^, Activated CD8^+^, and Regulatory T cells, as well as T follicular helper cells, Type 1 and Type 17 T helper cells differentiated from Activated CD4^+^ T cells.

Due to their unique cytokine profiles, differentiated classic subtypes play an important role in regulating immunological responses when CD4^+^ T cells are activated (14, 32). Th1 cells set cell-mediated immune responses, Th2 cells mediate humoral-mediated responses, Th17 cells mediate type 3 immune responses against extracellular pathogens (including some bacteria and fungi), and Tregs are essential for immune cell homeostasis and prevention of autoimmune diseases (14, 32). With the strong plasticity and heterogeneity of CD4^+^ T cells that tend to be an immune microenvironment which co-regulates by cytokines with different functions released by Th1/Th2/Th17/Treg cells, and the balanced relationship between cell subpopulations is not necessarily the key factor leading to an inflammatory disease, such as the “classic Th1/Th2 balance disease induction model” (33–35). Then, we examined the phenotypes of each subtype on titanium nanotubes of various diameters and their representative cytokines, such as: Th1(*IFN-γ*), Th2 (*IL-4*), Th17 (*IL-17A*、*TNF-α*、*IL-6*), Treg (*TGF-β1*、*IL-10*) (36–38). *IL-4*, *TGF-β1*, and *IL-10* are anti-inflammatory, while *IFN-γ*, *IL-17A*, *TNF-α*, and *IL-6* are pro-inflammatory. The immunological properties of each subtype depend on these cytokines. The results showed that compared to large-diameter titanium nanotubes (200 nm), medium-diameter titanium nanotubes (100 nm) promoted the expressions of the transcription factors *Rorγt* (Th17), *Gata3* (Th2), and *Foxp3* (Treg) while inhibiting *T-bet* (Th1) expression. Most cytokines coincided with the expression trends of main transcription factors in each subtype, such as *IFN-γ*, *TNF-α*, *IL-6*, *IL-10*, *IL-17A*. *TNF-α* and *IL-6* were significantly reduced in NT40 and NT15 groups, while *IL-17A* was increased, consistent with the expression of *Rorγt* (Th17), which supporting *IL-17A* as the primary cytokine produced by Th17 cells. In addition, CD4^+^ T cells and their cytokines regulate bone healing. According to Liu et al. (39), lymphocytes may inhibit mesenchymal stem cells (MSCs) survival and osteogenic differentiation by secreting pro-inflammatory cytokines *IFN-γ* and *TNF-α*. Croes et al. (13) found that pro-inflammatory Th17 cells have the greatest impact on osteogenesis, with *IFN-γ* promoting and *TGF-β* inhibiting this process. This study further supports that *IFN-γ* and *TNF-α* may inhibit osteogenesis, as their expressions were reduced in the NT40-100 nm and NT15-30 nm groups, aligning with the osteoclast factor *RANKL* but opposing the osteogenic factor *OPG*. Similarly, the findings support a pro-osteogenic role for *TGF-β1*, which showed increased expression on titanium nanotubes compared to the P group, consistent with *OPG* expression. For angiogenesis, VEGFA stands out as the most effective factor in the VEGF family, significantly promoting neovascularization (44). Ang-2 complements VEGFA by responding to its sprouting signal, working synergistically to support angiogenesis (40). Under hypoxic conditions, HIF-1α further enhances VEGFA and Ang-2 expression, helping to regulate angiogenesis. In this study, we found that NT15-30 nm nanotubes are optimal for inducing activated T cells to express angiogenic factors *VEGFA* and *Ang-2*, while NT40-100 nm nanotubes better support HIF-1α expression. This suggests that NT15-30nm nanotubes may regulate *VEGFA* and *Ang-2* expression independently of the HIF-1α pathway. Overall, the results reveal that medium-diameter titanium nanotubes promote activated T cells to express osteogenic genes, while small-diameter nanotubes encourage angiogenic gene expression. Both morphologies also promote anti-inflammatory and inhibit pro-inflammatory gene expressions in T cells, except for IL-17A.

Bioinformatics analysis of public datasets showed that *IL-17A* is a key immune factor for healthy implants. Six cytokines comprise the powerful pro-inflammatory IL-17 category, and the most typical cytokine is *IL-17A*, also known as *IL-17* (41). Then, T cells were sequenced, and comparable findings were obtained. Titanium nanotubes regulate immune function of T cells with *Ccl7* and *Cxcl2* chemokines. Previous research found that *IL-17* might stimulate these molecules which recruit neutrophils, macrophages, and lymphocytes to jointly regulate immunity and heal tissue damage (41). These two chemokines are concentrated in the IL-17 signaling pathway mediated by *IL-17A/F*, which governs Th17 cell development and cytokine interactions. Previous investigations have shown that Th17-derived soluble factors *IL-17A* and *IL-17F* up-regulate osteogenic marker expression in MSCs, with *IL-17A* synergistically affecting bone morphogenetic protein 2 (13). Yu et al. (42) observed that bone-mimicking hydroxyapatite (HAp) nanorods (HAp-100) cause T cells to release the *IL-22* most and enhance bone marrow stromal cell osteogenesis and activate the JAK1/STAT3 signaling pathway in conditioned medium. Anti-*IL-22* neutralizing antibodies can resist these effects. Additionally, *IL-22* is one of the major cytokines secreted by the Th17 subtype (43). Thus, through the IL-17A signaling pathway, T cell-derived *IL-17A* may be a key immune factor that governs T cell differentiation and cytokine secretion with titanium nanotubes, particularly those with the medium diameter (100 nm), as confirmed by gene and protein levels. However, to determine whether the secretion of *IL-17A* promoted by 100-nm titanium nanotubes enhances bone remodeling through the downstream targets ERK, NF-*_K_* B, or MAPK of the IL-17A/F signaling pathway, we need to design more experiments.

## 5 Conclusion

This research showed that titanium nanotubes with different diameters modulate T cell immunoactivity in different ways. In particular, 100-nm and 30-nm titanium nanotubes enhance T cell differentiation into Th17, Th2, and Treg subtypes, creating an anti-inflammatory, pro-osteogenic, and pro-angiogenic microenvironment. T-cell-derived IL-17A appears to play a key regulatory role in this process, laying the groundwork for future studies on adaptive immunity at the nanotopography interface.

## Data Availability

The datasets presented in this study can be found in online repositories. The names of the repository/repositories and accession number(s) can be found below: https://www.ncbi.nlm.nih.gov/, GSE106090 https://www.ncbi.nlm.nih.gov/, GSE178351 https://www.ncbi.nlm.nih.gov/, GSE57631 https://www.ncbi.nlm.nih.gov/, PRJNA1069315.

## Supplementary material

Supplementary Figure 1 The GSE106090, GSE57631, and GSE178351 datasets were merged, and the distribution differences were exhibited before and after removing batch effects. **(A, B)** Boxplots; **(C, D)** Density Distribution; **(E, F)** Umap Distribution. Reproduced from ref. (23) with permission from Immunity, inflammation and disease. copyright 2024.

Supplementary Figure 2 Six clusters were obtained by PPI network. **(A)**. Cluster 1, Score: 39.023; **(B)**. Cluster 2, Score: 15.486; **(C)**. Cluster 3, Score: 11.462; **(D)**. Cluster 4, Score: 4; **(E)**. Cluster 5, Score: 3.333; **(F)**. Cluster 6, Score: 3. Blue, light green, dark green, orange, pink and gray blue represented T-cell-related hub genes, inhibitory factors, stimulatory factors, receptors, chemokines, and MHC, respectively.
