# Supplementary figures and images for: Titanium nanotubes modulate immunophenotyping and cytokine secretion of T cells via IL-17A: a bioinformatic analysis and experimental validation
Source: Front Immunol. 2025 Jan 7;15:1381158. doi: 10.3389/fimmu.2024.1381158 (PMC11747796; doi:10.3389/fimmu.2024.1381158)

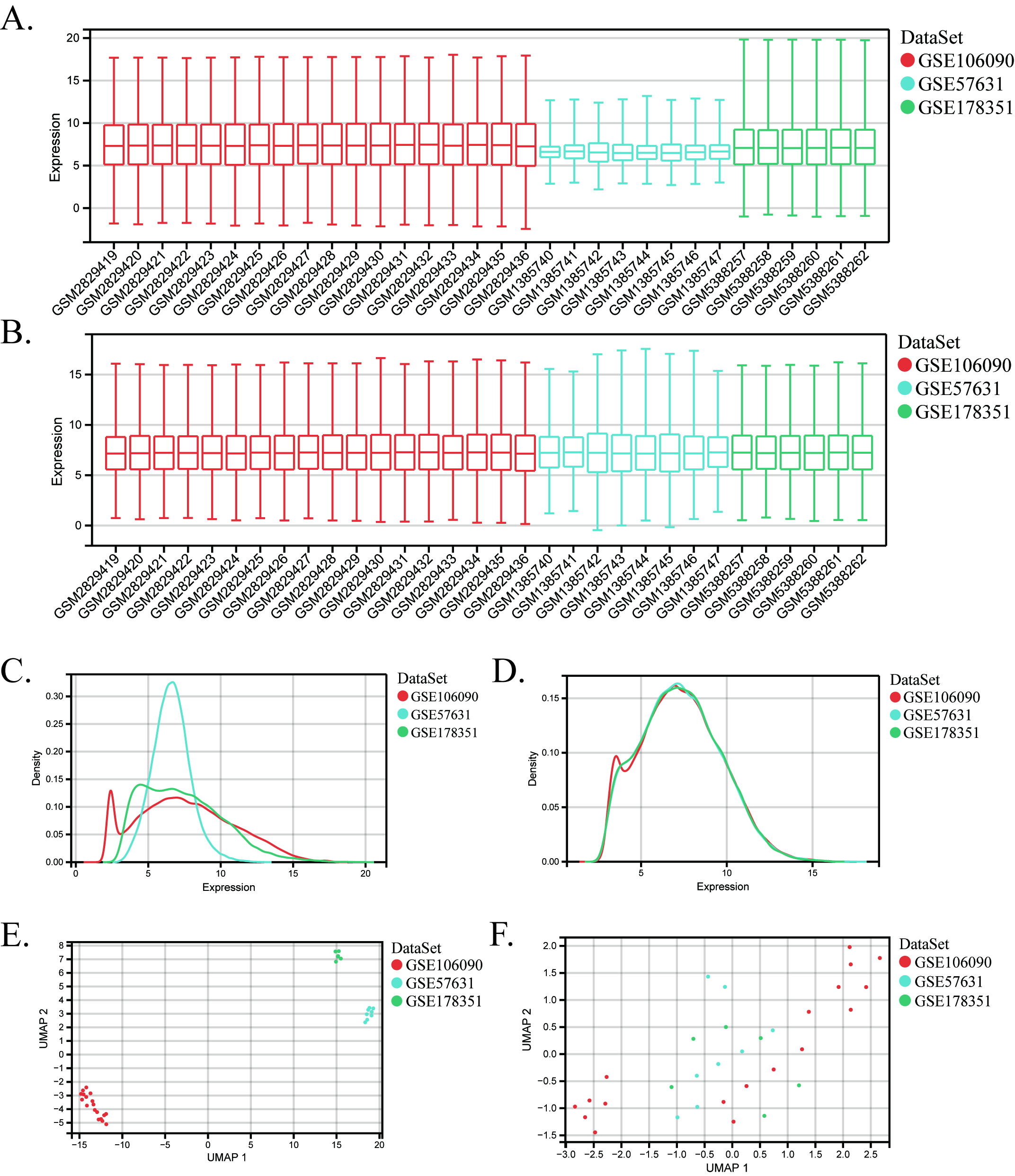

Supplement: Supplementary Figure 1 — The GSE106090, GSE57631, and GSE178351 datasets were merged, and the distribution differences were exhibited before and after removing batch effects. (A, B) Boxplots; (C, D) Density Distribution; (E, F) Umap Distribution. Reproduced from ref. (23) with permission from Immunity, inflammation and disease. copyright 2024. [file Image1.tif]

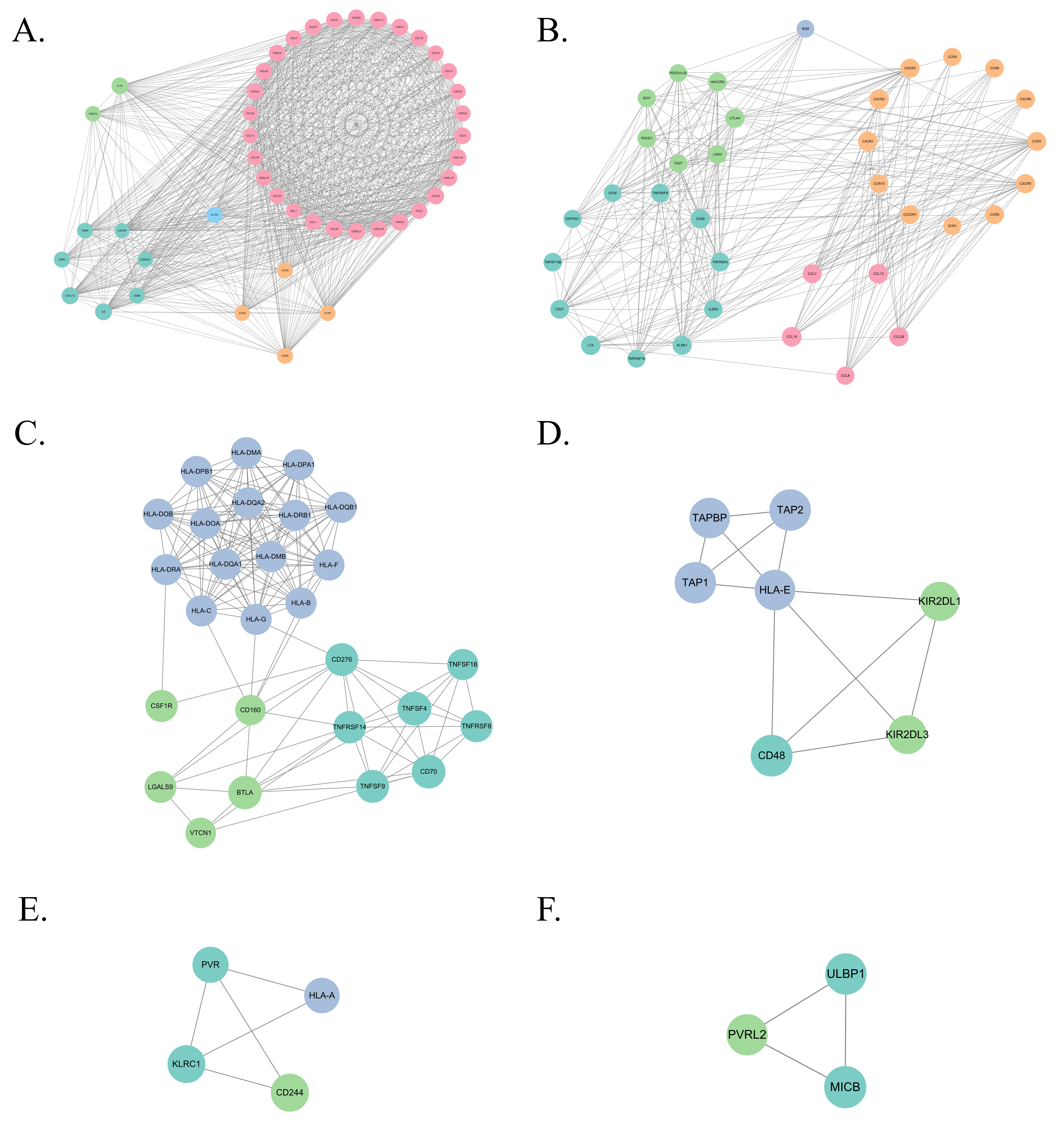

Supplement: Supplementary Figure 2 — Six clusters were obtained by PPI network. (A). Cluster 1, Score: 39.023; (B). Cluster 2, Score: 15.486; (C). Cluster 3, Score: 11.462; (D). Cluster 4, Score: 4; (E). Cluster 5, Score: 3.333; (F). Cluster 6, Score: 3. Blue, light green, dark green, orange, pink and gray blue represented T-cell-related hub genes, inhibitory factors, stimulatory factors, receptors, chemokines, and MHC, respectively. [file Image2.tif]
