# Supplementary material for: Titanium nanotubes modulate immunophenotyping and cytokine secretion of T cells via IL-17A: a bioinformatic analysis and experimental validation
Source: Front Immunol. 2025 Jan 7;15:1381158. doi: 10.3389/fimmu.2024.1381158 (PMC11747796; doi:10.3389/fimmu.2024.1381158)
Supplement: Supplementary file 3 [file Table1.docx]

**Supplementary Table 1. Different diameter, roughness, and contact angle of titanium nanotubes**

|  | P | NT15 | NT40 | NT70 |
| --- | --- | --- | --- | --- |
| Diameter | / | 30.59±4.99nm | 102.95±7.64nm | 203.47±8.70nm |
| Ra | 26.03±13.67nm | 77.50±3.50nm | 114.33±8.08nm | 128.33±13.05nm |
| Rq | 35.80±17.11nm | 101.10±5.12nm | 144.67±10.02nm | 161.00±16.70nm |
| Contact angle | 85.90±5.41° | 2.13±3.30° | 12.10±4.23° | 3.80±2.94° |
