# Supplementary material for: Titanium nanotubes modulate immunophenotyping and cytokine secretion of T cells via IL-17A: a bioinformatic analysis and experimental validation
Source: Front Immunol. 2025 Jan 7;15:1381158. doi: 10.3389/fimmu.2024.1381158 (PMC11747796; doi:10.3389/fimmu.2024.1381158)
Supplement: Supplementary file 4 [file Table2.docx]

**Supplementary Table 2. Primer sequences**

| Gene | Primer sequence（5′–3′） | Length（bp） |
| --- | --- | --- |
| *T-bet* | Forward: TCTGTCGAACCAGTATCCTGTCTCC  Reverse: CTTCACGCTCACTGCTCGGAAC | 142 |
| *Gata3* | Forward: TACATGGACCCGGCACAGTACC  Reverse: ACCGTAGCCCTGACCGAGTTTC | 113 |
| *Rorγt* | Forward: CTACGCCTGGAGGACCTTCTACG  Reverse: CTCCCACATCTCCCACATTGACTTC | 96 |
| *Foxp3* | Forward: GCAGCTCCGGCAACTTTTC | 101 |
|  | Reverse: CTTGTCTGAGGCAGGCTGGAT |  |
| *RANKL* | Forward: CGTACCTGCGGACTATCTTCA  Reverse: TTGGACACCTGGACGCTAAT | 195 |
| *OPG* | Forward: ATTATACCGAATTGGCTGAGTGTT  Reverse: GGTCAATGTCTTGGATGATCTTCT | 184 |
| *VEGFA* | Forward: TTACTGCTGTACCTCCACCAT  Reverse: CAGGACGGCTTGAAGATATACTC | 188 |
| *ANG-2* | Forward: ATCTTGTCTTGGCCTCAGCC  Reverse: AATGTGTAGCTGCAGGGTCC | 100 |
| *HIF-1α* | Forward: AATCTGAGGACACGAGCTGC  Reverse: GCTGCCGAAGTCCAGTGATA | 110 |
| *IL-4* | Forward: CTTACGGCAACAAGGAACACC  Reverse: AGACCGCTGACACCTCTACA | 158 |
| *IL-10* | Forward: GTGGAGCAGGTGAAGAATGATT | 102 |
|  | Reverse: CACGTAGGCTTCTATGCAGTTG |  |
| *TGF-β1* | Forward:GGACCGCAACAACGCAATCTATG | 96 |
|  | Reverse:TCTGGCACTGCTTCCCGAATG |  |
| *IFN-γ* | Forward:ACCCACAGATCCAGCACAAAGC | 83 |
|  | Reverse:CCGCTTCCTTAGGCTAGATTCTGG |  |
| *TNF-α* | Forward: CTCAAGCCCTGGTATGAGCC | 130 |
|  | Reverse: CTCCAAAGTAGACCTGCCCG |  |
| *IL-6* | Forward: GACTTCCAGCCAGTTGCCTT | 112 |
|  | Reverse: CTGGTCTGTTGTGGGTGGTAT |  |
| *IL-17A* | Forward:CCATGTGCCTGATGCTGTTG | 104 |
|  | Reverse:GTTATTGGCCTCGGCGTTTG |  |
| *GAPDH* | Forward: ACGGCAAGTTCAACGGCACAG  Reverse: GAAGACGCCAGTAGACTCCACGAC | 149 |
